# Massive attack by honeybees in a German shepherd dog: description of a fatal case and review of the literature

**DOI:** 10.1186/1678-9199-20-55

**Published:** 2014-12-13

**Authors:** Mudassar Niaz Mughal, Ghazanfar Abbas, Muhammad Saqib, Ghulam Muhammad

**Affiliations:** Department of Clinical Medicine and Surgery (CMS), Faculty of Veterinary Sciences (FVS), University of Agriculture Faisalabad (UAF), Faisalabad, 38040 Punjab Pakistan

**Keywords:** German shepherd, Honey bee, Sting

## Abstract

In the present study, a fatal case caused by honeybee (*Apis cerana*) stings was documented in a female German shepherd dog that was presented at the Veterinary Teaching Hospital, University of Agriculture Faisalabad, Pakistan. Characteristic clinical signs included hematuria, hematemesis, incoordination and convulsions along with evidence of massive honeybee attack supported the diagnosis of envenomation. The dog was treated with dexamethasone and diphenhydramine, but it did not respond to therapy and died. This outcome could be avoided if we had a bee antivenom available for treating envenomated patients.

## Background

Although honeybees usually do not sting until they are provoked, their stings threaten lives of humans and other animals [1, 2]. The literature provides few studies related to the clinicopathological picture of animals stung by insects, especially honeybees. In Canada and USA, the number of deaths attributed to bee and wasp stings annually are one and more than thirty individuals, respectively [3]. In Brazil, the prevalence of Africanized honeybee stings has shown significant increase year after year. For example, 4,774 and 10,026 sting cases were registered respectively in 2006 and 2012 in that country [4]. Although notification is not compulsory, each year approximately 15,000 attacks occur, causing an estimated number of 140 deaths in Brazil [4–6]. This is usually a problem of rural areas, public parks and groves where bee swarms are found hanging from tree branches [1]. Introduction of bees in urban areas is also posing threats to humans and animals. While migrating, swarms may also attack livestock populations. The present case report describes a fatal case of honeybee stings in a German shepherd dog along with a review of the literature.

## Case presentation

**Figure 1:**
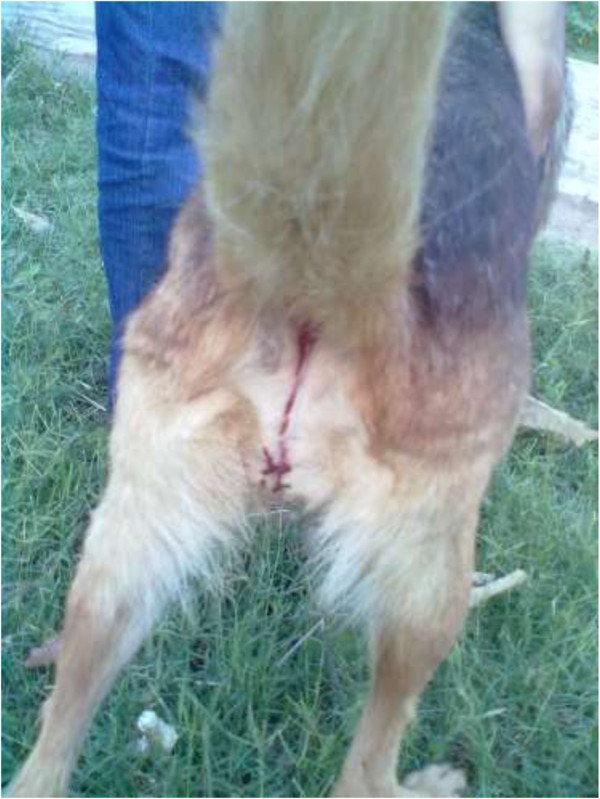
**German shepherd dog affected by massive honeybee stings.** Observe the bloody diarrhea and hematuria.

A 2.5-year old unspayed female German shepherd dog weighing 22 kg was presented to the Veterinary Medical Teaching Hospital (VMTH), Department of Clinical Medicine and Surgery, University of Agriculture Faisalabad, Pakistan, with bloody vomiting, hematuria and incoordination immediately after a massive attack of honeybees that stung its face, tongue, ears, fore and hind limbs, teats and external genitalia (Figure 1).

Clinical examination carried out one hour after the stings revealed slightly elevated rectal temperature (39.6°C), pale and congested mucous membranes, tachycardia (160 beats/minute), tachypnea (60 breaths/minute), staggering gait, seizures, hematuria and bilateral mydriasis. Face, tongue, external genitalia and the four legs were markedly swollen and had multiple embedded stingers. Erythema was prominent at the sting sites. The significant biochemical alterations were leukopenia, anemia, elevated liver enzymes, serum creatine kinase, creatinine and blood urea nitrogen (Tables 1 and 2). Urinalysis showed hematuria and increased specific gravity.

**Table 1:** **Hematological values of a female German shepherd dog attacked by honeybees**

Parameters	Values taken before and after initiation of treatment		
	Day 1	Day 2	Reference values*
Red blood cells (RBC; × 10^12^ g/L)	3.9	2.2	5.5-8.5
Packed cell volume (PCV; %)	26	22	37-55
Hemoglobin (Hgb; g/dL)	9	5	12-18
Mean corpuscular volume (MCV; fL)	35	29	60-77
Mean corpuscular hemoglobin (MCH; pg)	13	11	19.5-24.5
Mean corpuscular hemoglobin concentration (MCHC; 10 g/L)	26	23	32-36
White blood cells (WBC; × 10^ς^ L)	4	3	6-17
Neutrophils (%)	40.2	32.7	60-70
Lymphocytes (%)	4.8	1.3	12-30
Monocytes (%)	2	1	4-10
Basophils (%)	12.1	13	Rare
Eosinophils (%)	40.9	52	2-10

*The Merck Veterinary Manual [7].

**Table 2:** **Serum biochemistry values of a female German shepherd dog attacked by honeybees**

Parameters	Values taken before and after initiation of treatment		
	Day 1	Day 2	Reference values*
Aspartate aminotransferase (μ/L)	100	122	8.9-49
Alanine aminotransferase (μ/L)	110	129	8.2-57
Alkaline phosphatase (μ/L)	150	170	10.6-101
Creatine kinase (μ/L)	160	188	14-120
Creatinine (mg/dL)	2.5	3.2	0.5-1.6
Blood urea nitrogen (mg/dL)	38	52	8.8-26

*The Merck Veterinary Manual [7].

The patient was immediately treated with intravenous crystalloid bolus (40 mL/kg), dexamethasone (0.5 mg/kg) and diphenhydramine (1.3 mg/kg) and monitored for the next 4–5 hours at the VMTH. Approximately 150 stingers were removed manually from the body. The patient showed a slight positive response to treatment and was discharged with the advice to repeat the same treatment after 12 hours. Next day, the dog was again presented in comatose position having pale mucous membranes, subnormal body temperature, increased capillary refill time and severe bradycardia. Results of hematology and serum biochemistry analysis (Tables 1 and 2) were similar to those of initial presentation. Before any treatment started, the dog died. Necropsy was planned, but the guardian did not allow. Diagnosis was based on clinical signs, gross findings, identification and observation of attacks of bees.

## Discussion

Honeybees are herbivores that feed on nectar and pollen, which belongs to the family Apoidea, class Insecta and order Hymenoptera. Three indigenous (*Apis dorsata*, *Apis cerana*, and *Apis florea*) and one foreign (*Apis mellifera*) species of honeybees are found in Pakistan [8].

Honeybee venom is a transparent acidic substance composed of different enzymes, proteins and amines that has the ability to produce toxic and allergic reactions in the body of affected individuals [9]. Bee venoms may be used as an antibiotic agent and to immunize individuals against certain infectious diseases [1]. The main constituents of bee venom are melittin and phospholipase A_2_, although many other substances are also present. These substances cause hemolysis, rhabdomyolysis, degeneration and necrosis of kidney tubules leading to the organ failure [5, 10]. There are certain factors that regulate the proportion and the toxicity of honeybee venoms, which include weather condition, age and types of flowers used by bees for honey production [11, 12]. Bee venom is heat resistant and retains its toxicity for long periods.

Melittin, that accounts for half of the weight of dried venom, has cytotoxic, hemolytic and cardiotoxic properties [1, 13]. It also acts like a detergent and is responsible for release of histamine and local pain at the sting site [6]. Catecholamine (released from melittin) along with phospholipase A_2_ is responsible for intravascular hemolysis [14]. Phospholipase A_2_ (12% of dry venom weight) is a well known allergen. Hyaluronidase present in the venom, also known as “spreading factor”, is responsible for changing the permeability of cell membranes, thus allowing the other components of venom to penetrate into host tissues.

Humans and other animals are accidently stung by bees if they are disturbed. The clinical signs associated with their sting may vary from mild to severe depending upon some factors including type and quantity of venom, stinging site and number of stings along with sensitivity of the victim [5]. Most lesions affect exposed parts of body such as face and limbs, since dense fur protects major areas of the animal body [14]. The estimated lethal dose for humans and mammals is about 500 stings/adult and 20 stings/kg [15], respectively. In the literature, a wide range of numbers of bee stings (60–2460) has been related with the death of dogs. Even a single sting may lead to death of an individual [16].

The possible outcomes caused by bee stings include delayed hypersensitivity, anaphylactic shock, local and systemic reactions. Immune-mediated secondary hemolytic anemia may be observed in affected dogs after multiple stings [17]. Most deaths due to bee stings are the result of anaphylactic shock after delayed hypersensitivity reaction mediated by IgE antibodies. However, massive envenomation may also kill those individuals that are not allergic to bee venom. In one study, bumblebee sting associated with anaphylactic shock has been reported in a dog [18]. Anemia, pale and congested mucous membranes, tense abdomen, obtundation, generalized seizure and episodic cardiac arrest were prominent findings in the affected animal. The dog was treated symptomatically along with supportive therapy and recovered completely after six weeks [18]. Similar findings were presented in our report so the possibility of fatal honeybee sting associated with anaphylactic shock cannot be overlooked.

Honeybees can sting only once, since their stingers remain attached to the skin of victims. Contrarily, bumblebee stingers cannot be found at the affected site, because these bees have the ability to retract their stinger [18]. Venom of Hymenoptera bees can directly cause neurotoxicity, this is the reason for ataxia and facial paralysis in a dog victim of massive stings [19]. Neurotoxicity is due to the presence of apamin in bee venom, which acts on the spinal cord [14]. According to a retrospective study that included 19 dogs that died due to envenomation caused by multiple Africanized bee stings, head and neck are the most severely affected portions of the body, whereas sting-associated edema, hyperemia and erythema were found all over the body. Major pathological findings in affected dogs were muscular necrosis, hemorrhage and congestion of different organs, splenomegaly, dark red urine, kidney and lungs [20]. Presence of jaundice and different pigments such as myoglobin and hemoglobin inside the kidney tubules and bile ducts indicated that red blood cells and muscle cells had been destroyed by melittin and phospholipase A_2_ [11].

Results of various studies on honeybee venoms concluded that melittin has a potential role in order to activate bradykinin (BK) release as well as hemolytic pathway in the body of victim. Melittin has ability to attach and generate transient openings on the surface of red blood cells through which approximately 40 hemoglobin molecules can flee easily resulting in decreases level of packed cell volume [21, 22].

In another report on three cases of Africanized honeybee stings in cattle, it is recommended that sting-associated lesions in affected animals, especially scar tissue retraction of ears, should be differentiated from photosensitization on the basis of history and clinic-pathological lesions [23]. Histological examination of skin of the affected animals revealed findings similar to those observed in horses affected by chronic photosensitization caused by *Brachiari humidicola* [24]. The cattle was developing subcutaneous edema, necrosis and detachment of superficial skin layer, and showed complete regeneration of the epithelium after 45 days after the stings.

Necropsy of animals envenomated by hymenopterans usually does not reveal pathognomonic lesions whereas pathological changes are general even after fatal anaphylactic shock [25]. In suspected fatal cases of anaphylactic shock, particular attention should be given to the larynx of victim regarding the presence of edema, hyperemia and hemorrhages [26]. Unfortunately, we do not have the bee antivenom available. Therefore, a conservative treatment including administration of antihistaminic agents and corticosteroids both parentally and topically was adopted [14]. The most important step regarding treatment is to pull out the stingers as soon as possible to avoid the risk of further spread of venom into the body [1].

## Conclusion

In the present communication, we report a rare case of honeybee sting associated with death in a German shepherd dog.

### Ethics committee approval

The management of this case was in agreement with the guidelines of the Ethics Committee of the University of Agriculture, Faisalabad, Pakistan.
